# Effects of all-trans retinoic acid and oxygen concentration on the proliferation and differentiation of subcutaneous adipocytes in yak

**DOI:** 10.3389/fvets.2025.1643818

**Published:** 2025-08-26

**Authors:** Sibinuer Yasenjian, Su Shan, Yuqi Zhe, Yuqing Zhang, Zhixin Chai

**Affiliations:** ^1^Key Laboratory of Qinghai-Tibetan Plateau Animal Genetic Resource Reservation and Utilization, Chengdu, China; ^2^Sichuan Qinghai-Tibet Plateau Herbivore Livestock Engineering Technology Center, Chengdu, China

**Keywords:** yak, subcutaneous adipocytes, all-trans retinoic acid, proliferation, differentiation, hypoxia adaptation

## Abstract

In this study, we investigated the mechanism by which all-trans retinoic acid (ATRA) regulates the hypoxic adaptation of yak subcutaneous adipocytes through a dose-dependent regulation. The effects of different concentrations of ATRA (10 nM−10 μM) on cell proliferation and differentiation, lipid metabolism and key gene expression were analyzed by simulating a hypoxic environment (1% O_2_) *in vitro*. In the hypoxia model, ATRA showed biphasic regulation: the hypoxic environment inhibited cell survival, while low concentrations of ATRA (10–100 nM) significantly enhanced hypoxic cell survival and repaired lipid droplet accumulation by activating the PCNA/PPAR-γ signaling axis; while high concentrations of ATRA (1–10 μM) induced apoptosis and inhibited differentiation by upregulating BAX and inhibited differentiation, and its toxic effects were exacerbated by the hypoxic environment. In addition, a certain concentration (10–100 nM) of ATRA antagonized the hypoxia-induced HIF-1α pathway and regulated hypoxia-induced metabolic reprogramming via PCNA/PPAR-γ. The study revealed a dose-dependent bidirectional regulatory mechanism of ATRA in the hypoxic adaptation of yak adipocytes, which provides a new perspective for analyzing the energy metabolism strategy of plateau species.

## 1 Introduction

The yak (*Bos grunniens*), as a rare species unique to the Qinghai-Tibet Plateau, has evolved unique hypoxia adaptation mechanisms through long-term natural selection, enabling it to tolerate low temperatures, hypoxia, and strong ultraviolet radiation in high-altitude environments. Its physiological characteristics are not only crucial for maintaining the stability of the plateau ecosystem but also provide an ideal model for studying biological hypoxia adaptation mechanisms (1). The hypoxic environment of the plateau is not only an important factor influencing the occurrence and development of various diseases such as cancer but also imposes limitations on the survival of local organisms and the sustainable development of animal husbandry (2). Long-term exposure to a hypoxic environment profoundly affects cellular metabolism, proliferation, and differentiation. Therefore, an in-depth exploration of the cellular physiological and molecular adaptation mechanisms of organisms to hypoxic environments, the search for substances that can assist animals in better adapting to hypoxia, and the establishment of a comprehensive hypoxia adaptation and body protection system are of great significance for promoting the development of plateau animal husbandry and improving human quality of life.

Adipose tissue is not only the largest reservoir of chemical energy in the body but also a dynamic endocrine organ that participates in oxygen sensing and metabolic homeostasis regulation by secreting adipokines such as leptin and adiponectin (3, 4). Mammalian adipose tissue is classified into subcutaneous fat (SF), intramuscular fat, and visceral fat based on its distribution. SF's primary functions include thermal insulation, maintaining constant body temperature, storing energy, and metabolic balance (5). SF is also a critical factor for high-altitude animals to resist cold environments and for energy metabolism. Studies have found that low temperatures and hypoxic conditions can induce increased expression of uncoupling protein 1 (UCP1) and uncoupling protein 3 in skeletal muscle and subcutaneous adipose tissue, along with metabolic changes, indicating the importance of SF for energy metabolism and resistance to cold environments under low temperature and hypoxia conditions (6). Hypoxia-inducible factor (HIF) is the main transcription factor mediating oxygen receptors in tissues and organs. HIF can induce diseases by regulating lipid synthesis, fatty acid metabolism, and lipid droplet formation. Research on the regulation of transcriptional regulators mediated by hypoxic stress and the regulation of adipocyte development and lipid metabolism has revealed the potential mechanisms underlying hypoxia-induced changes in adipocyte metabolic pathways (7). Under hypoxic conditions, activation of the HIF-1α pathway induces upregulation of the expression of key enzymes of glycolysis and enhances the efficiency of free fatty acid release through PPARγ-mediated lipid mobilization mechanism. This metabolic switch has a dual effect: in the short term by enhancing the rapid energy supply of glycolysis, and in the long term by promoting the remodeling of adipose tissue microvascular network through the up-regulation of vascular endothelial growth factor (VEGF) expression, which systematically improves the efficiency of tissue oxygenation (8). Notably, the hypoxic environment itself forms a feedback regulation, ultimately affecting the overall functional state of adipose tissue by regulating key processes such as adipocyte differentiation, lipid metabolism balance, and the intensity of inflammatory responses.

All-trans retinoic acid (ATRA) is one of the active metabolites of vitamin A and plays a crucial regulatory role in physiological processes such as cell growth, differentiation, metabolism, and apoptosis (9). Studies have shown that ATRA can regulate the expression of target genes by binding to retinoic acid receptors (RAR) and retinoid X receptors, thereby influencing cellular biological behavior. In adipocytes, ATRA can regulate adipocyte differentiation and lipid metabolism. For example, ATRA can inhibit the differentiation of 3T3-L1 preadipocytes into mature adipocytes and modulate the expression of adipokines such as adiponectin and leptin in adipocytes, thus affecting the function of adipose tissue (10). Meanwhile, ATRA exhibits dose-dependent regulatory characteristics in adipocyte differentiation. Research has shown that when the concentration of ATRA exceeds 1 μM, it significantly inhibits the lipid deposition process in 3T3-L1 preadipocytes (10, 11). However, this inhibitory effect shows cell-specific differences: in the Ob1771 cell line, low concentrations of ATRA (10 nM) instead promote adipogenesis (12). Although existing studies have revealed the role of ATRA's dose-dependent regulatory mechanisms in adipocyte differentiation and metabolism, its regulatory effects and mechanisms on hypoxia adaptation in subcutaneous adipocytes of yaks, a species unique to high-altitude environments, remain unexplored. Investigating these interactions under this special physiological environment is of great significance for understanding the energy metabolism characteristics of yaks.

This study aims to investigate the effects of ATRA on the hypoxia adaptation of yak subcutaneous adipocytes and its molecular mechanisms. By culturing yak subcutaneous adipocytes *in vitro* and simulating a hypoxic environment, the study examines the effects of ATRA on the proliferation, differentiation, lipid metabolism, and hypoxia-related gene expression of yak subcutaneous adipocytes under hypoxic conditions. At the same time, it delves into the signaling pathways through which ATRA regulates the hypoxia adaptation of yak subcutaneous adipocytes, providing new insights and theoretical foundations for elucidating the survival mechanisms of yaks in hypoxic environments.

## 2 Materials and methods

### 2.1 Animal materials

The animals used in the experiment were 48-month-old male yaks of similar weight male Maiwa yaks of similar body weight, which were grazed in highland pastures until adulthood, and subcutaneous adipose tissue of yaks was harvested in October–November, and the abdominal subcutaneous adipose tissue was collected rapidly during the yak slaughtering process, and then rinsed with sterilized phosphate-buffered saline (PBS) until the surface was free of blood stains (P/S; Gibco, Thermo Fisher Scientific) in 3% penicillin-streptomycin (P/S; Gibco, Thermo Fisher Scientific) and brought back to the sterile cell room. All animal experimental procedures were reviewed and approved by the Animal Care and Use Committee of the Southwest University for Nationalities and were conducted in accordance with the relevant guidelines and regulations established by the local animal ethics committee.

### 2.2 Isolation and culture of yak subcutaneous preadipocytes

**Figure 1:**
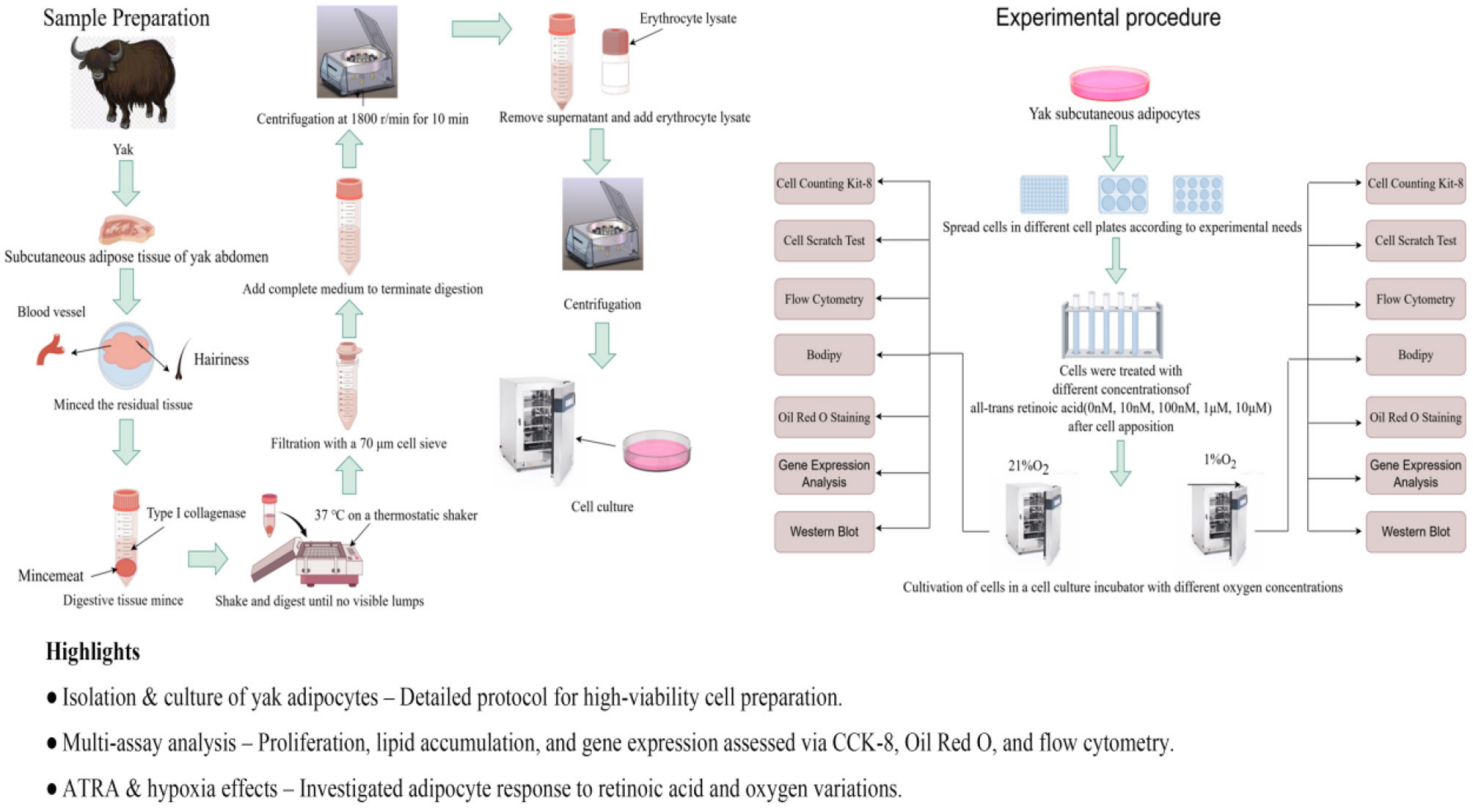
Experimental flow chart (by Figdraw). Highlights: • Isolation and culture of yak adipocytes–detailed protocol for high-viability cell preparation. • Multi-assay analysis–proliferation, lipid accumulation, and gene expression assessed via CCK-8, Oil Red O, and flow cytometry. • AIRA and hypoxia effects–investigated adipocyte response to retinoic acid and oxygen variations.

The subcutaneous adipose tissue was washed three times in PBS containing 5% penicillin-streptomycin-amphotericin B (Gibco, Thermo Fisher Scientific). The fascia was removed with scissors, and the tissue was then cut into 1–3 mm^3^ pieces using scissors. The tissue fragments were digested in a digestion solution (Collagenase Type I; Gibco, Thermo Fisher Scientific) at 180 rpm/min on a shaker for 2.5–3 h. The digested material was filtered through a 40 μm sterile cell strainer, and the filtrate was collected to terminate digestion. The filtrate was centrifuged at 1,200 rpm for 10 min, and the supernatant was discarded to obtain the cell pellet. The cells were resuspended in red blood cell lysis buffer and centrifuged under the same conditions. The resulting cell pellet was resuspended in culture medium and centrifuged at 1,000 rpm for 5 min. The supernatant was discarded, and this process was repeated twice. Finally, the cells were resuspended in growth medium and seeded into culture flasks. Differential adherence was used to purify the cells for 1 h, followed by culturing in an incubator (Thermo Fisher Scientific, Waltham, MA, USA) at 37 °C with 5% CO_2_. The culture medium was changed every 2 days until passaging or cryopreservation. The growth medium consisted of 89% DMEM (Gibco, Thermo Fisher Scientific) +10% fetal bovine serum (FBS; Gibco, Thermo Fisher Scientific) +1%penicillin-streptomycin (P/S; Gibco, Thermo Fisher Scientific). The differentiation medium was composed of growth medium supplemented with 100 μM oleic acid (Merck, Rahway, NJ, USA; Figure 1).

### 2.3 Treatment of yak preadipocytes

After cell seeding and attachment, the culture medium was replaced with ATRA (MedChemExpress, USA) containing medium. The ATRA stock solution (10 nM/L) was prepared in anhydrous DMSO, aliquoted, and stored at −80 °C protected from light. Working concentrations were diluted in complete culture medium. The culture medium was refreshed every 2 days to maintain final ATRA concentrations of 10 nM/L, 100 nM/L, 1 μM/L, and 10 μM/L for investigating subcutaneous adipocyte proliferation, differentiation, and apoptosis. All experimental groups and controls were cultured in parallel under two oxygen conditions (1% O_2_ vs. 21% O_2_). The 1% O_2_ condition was selected to simulate the hypoxic microenvironment of yak subcutaneous adipose tissue in high-altitude regions, as it closely approximates the physiological oxygen partial pressure (1–1.5 kPa) experienced *in vivo*. Preliminary experiments demonstrated weaker activation of the HIF pathway and lipid metabolism-related gene expression under 5% O_2_, justifying the final use of 1% O_2_ for hypoxia modeling.

### 2.4 Gene expression analysis

After treating the cells with different concentrations of ATRA, they were cultured in incubators with varying oxygen concentrations for 48 h. Total RNA was extracted from yak subcutaneous preadipocytes using TRIzol (Invitrogen, Thermo Fisher Scientific). The concentration and quality of the extracted RNA were measured using a spectrophotometer (NanoDrop One, USA). The extracted RNA was reverse transcribed using the PrimeScript™ RT Reagent Kit containing gDNA Eraser (Takara) and TB Green^®^ Premix Ex Taq™ II (Takara) for qPCR. The 2^−ΔΔCT^ method was used to calculate the relative mRNA expression levels of ATRA-related genes. GraphPad Prism 8.0 (GraphPad Software, La Jolla, CA, United States) was used to plot and analyze the significance of the results, with GAPDH serving as the internal reference gene. All primers used in this study were designed using Primer5 software (Table 1). The primers were synthesized by Chengdu Qingke Biotechnology Co, Ltd.

**Table 1:** Primer sequences detailed information.

**Genes**	**Gene ID**	**Forward primer (5^′^-3^′^)**	**Reverse primer (5^′^-3^′^)**
*GAPDH*	XM_014482068.1	CCACGAGAAGTATAACAACACC	GTCATAAGTCCCTCCACGAT
*PPAR-γ*	XM_005902846.1	CGTGGACCTTTCTATGATGGA	GCTCTTGGGAACGGAATG
*BAX*	NM_173894.1	GCCCTTTTGCTTCAGGGTTTC	TCAGACACTCGCTCAGCTTC
*RAR-α*	XM_005887847.2	TGATATCTCCAGACCGAGCC	TGAGGGGACGCGAGGAT
*HIF-1α*	NM_174339.3	TGAGGGGACGCGAGGAT	GCAATCGCAGAGGTCCAACT
*PCNA*	XM_005906528.2	CTCGTCTCATGTCTCCTTGGT	TGTCTTCATTGCCAGCACATT

### 2.5 BODIPY and oil red O staining

After treating the cells with different concentrations of ATRA, they were cultured in incubators with varying oxygen concentrations for 48 h. The culture medium was removed, and the cells were washed three times with PBS. Next, 4% paraformaldehyde (Biosharp, Hefei, Anhui, China) was added to each well for 30 min to fix the cells. After washing six times with PBS, Oil Red O or BODIPY (Invitrogen) working solution was added to each well, and the cells were stained in the dark for 30 min. The cells were then washed with PBS and incubated with DAPI (Solarbio, Beijing, China) staining solution in the dark for 10 min, followed by six washes with PBS. Finally, the cells were imaged under a microscope (Carl Zeiss, Oberkochen, Germany). After completion of oil red O staining, the dye was gently washed three times with PBS to remove unbound dye, followed by the addition of 1 ml of isopropanol per well and shaking at room temperature to dissolve the lipid droplet-bound dye. Two hundred microliters of the dissolved solution was transferred to a 96-well plate, and the absorbance was measured at 510 nm using an enzyme-linked immunoassay detector (Thermo Fisher Scientific).

### 2.6 Cell proliferation assay

#### 2.6.1 Cell counting kit-8 (CCK-8)

Subcutaneous preadipocytes were seeded into 96-well cell culture plates at a density of 8 × 10^3^ cells/well. Each well was supplemented with 100 μl of serum-containing basal medium [89% DMEM + 10% fetal bovine serum (FBS) + 1% penicillin-streptomycin] and cultured in an incubator at 37 °C with 5% CO_2_. When the cell confluency reached 50%, the medium was replaced with medium containing ATRA, with final concentrations of ATRA in the yak subcutaneous preadipocyte culture medium set at 10 nmol/L, 100 nmol/L, 1 μmol/L, and 10 μmol/L. Each experimental group had six replicates and was placed in cell culture incubators with different oxygen concentrations for continued culturing. At 0, 24, 48, 72, and 96 h, the culture medium was discarded, and each well was replenished with complete medium containing 10% CCK-8 (CCK-8, SparkJade). After further incubation at 37 °C for 1–4 h, the culture was terminated. The absorbance of each well was measured at a wavelength of 450 nm using an enzyme-linked detector (Thermo Fisher Scientific).

#### 2.6.2 Cell scratch test

Cells were seeded in 6-well culture plates at a density of 5 × 10^4^ cells/cm^2^, and each well was supplemented with 2 ml of serum-containing basal medium and cultured in an incubator at 37 °C with 5% CO_2_. Forty-eight hours after cell seeding, scratches were made using a 200 μL pipette tip aligned against the lid of the 6-well plate or a ruler, either parallel or perpendicular to the lines on the back. The serum-containing basal medium was discarded, and the cells were washed three times with PBS to remove the scratched-off cells. Serum-free medium containing ATRA was then added to achieve final concentrations of ATRA in the yak subcutaneous adipocyte culture medium of 10 nM/L, 100 nM/L, 1 μM/L, and 10 μM/L. Each concentration had three replicates and was placed in cell culture incubators with different oxygen concentrations for continued culturing. Cell migration was observed under a microscope at 0, 24, 48, and 72 h, and the results were photographed and saved.

#### 2.6.3 Flow cytometry

Cells were seeded in 6-well culture plates at a density of 5 × 10^4^ cells/cm^2^, and each well was supplemented with 2 ml of serum-containing basal medium and cultured in an incubator at 37 °C with 5% CO_2_. Forty-eight hours after cell seeding, the medium was replaced with medium containing ATRA to achieve final concentrations of ATRA in the yak subcutaneous adipocyte culture medium of 10 nM/L, 100 nM/L, 1 μM/L, and 10 μM/L. Each concentration had three replicates, and a control group was set up. These were placed in cell culture incubators with different oxygen concentrations for continued culturing. After 48 h, both the control group and the ATRA-treated subcutaneous preadipocytes were collected. Cells were double-stained with Annexin-V/PI (UElandy), data were acquired using a flow cytometer, and analyzed with Flowjo software. The apoptosis rate was determined by counting the number of cells in the apoptosis region.

### 2.7 Western blot analysis of protein expression levels of enzymes related to ATRA synthesis

Cells were washed twice with pre-cooled phosphate-buffered saline to remove residual medium, and then lysed on ice for 30 min in RIPA lysis buffer containing 1% protease inhibitor. The lysates were centrifuged at high speed (12,000 rpm, 15 min, 4 °C), and the supernatant was collected. Protein concentration was quantified using the BCA method (Pierce™ BCA Protein Assay Kit, Thermo Scientific). Equal amounts of protein samples (20 μg per well) were denatured with 5× SDS loading buffer and separated by 10% SDS-PAGE. Electrophoresis was performed at a constant voltage of 80 V for the stacking gel and 120 V for the separating gel. Proteins were then transferred to PVDF membranes (Merck Millipore) using a wet transfer system (Trans-Blot^®^ Turbo™, Bio-Rad) at a constant current of 250 mA. After transfer, the PVDF membranes were blocked with TBST buffer containing 5% skim milk at room temperature for 1 h. The membranes were incubated overnight at 4 °C with the following primary antibodies: PCNA (HUABIO, H651304031, rabbit monoclonal, 1:5,000) PPARγ (Proteintech, 16643-1-AP, rabbit monoclonal, 1:1,000), RAR-α (Proteintech, 10331-1-AP, rabbit monoclonal, 1:300), HIF-1α (Thermo Fisher, sc-13515, mouse monoclonal, 1:800), BAX (Proteintech, 50599-2-Ig, rabbit monoclonal, 1:2,000), and GAPDH as the internal reference (Proteintech, mAI-516, mouse monoclonal, 1:50,000). After washing the membranes three times with TBST (10 min per wash), corresponding species-specific HRP-conjugated secondary antibodies (goat anti-mouse IgG-HRP, invitrogen-31430, 1:10,000; goat anti-rabbit IgG-HRP, Proteintech-SA00001, 1:10,000) were added and incubated at room temperature for 1.5 h. Enhanced chemiluminescence substrate (ECL Prime Western Blotting Detection Reagent, GE Healthcare) was used for visualization, and signals were captured using a chemiluminescence imaging system (ChemiDoc™ MP Imaging System, Bio-Rad). Gray-scale values were analyzed using Image Lab 6.0 software. The expression levels of target proteins were normalized to GAPDH as an internal reference, and the experiment was repeated three times to ensure data reproducibility.

### 2.8 Data statistical analysis

The 2^−ΔΔCT^ method was used to convert the Ct values from qPCR into relative gene expression levels. Data analysis was performed using GraphPad Prism 8 software: comparisons between two groups were conducted using an independent sample *t*-test, and comparisons among multiple groups were analyzed using one-way analysis of variance (ANOVA), with multiple comparisons performed using the Duncan method. Data are expressed as mean ± stanard error of the mean (SEM). Significance levels were defined as ^*^p < 0.05, ^**^p < 0.01, ^***^p < 0.001, and ns indicates no significant difference.

## 3 Results

### 3.1 Effects of ATRA on the proliferation ability of yak subcutaneous adipocytes

**Figure 2:**
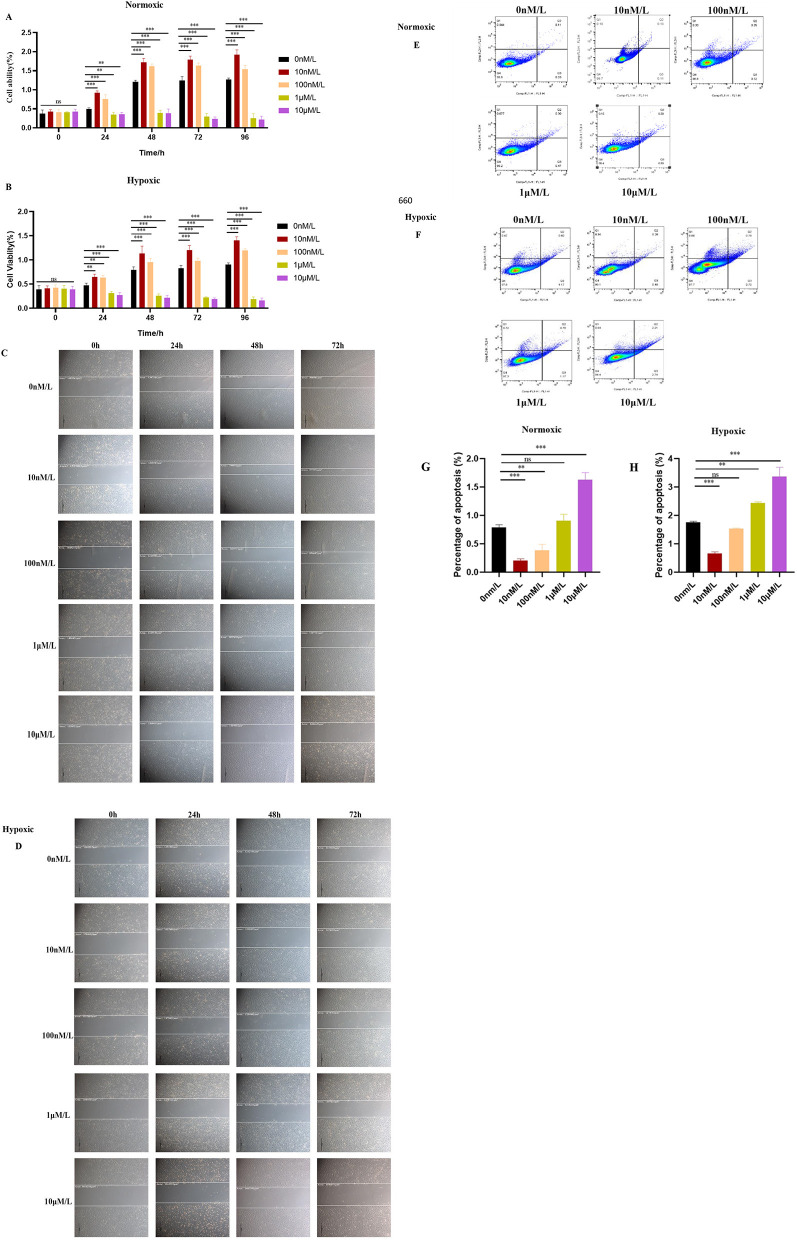
Effects of ATRA on the proliferation ability of yak subcutaneous adipocytes: **(A, B)** Cell viability of cells in normoxic and hypoxic groups treated with different concentrations of ATRA at 0, 24, 48, and 72 h. Data are presented as mean ± SEM for three independent experiments. Significance levels were defined as **p < 0.01, ^***^p < 0.001, and ns indicates no significant difference. **(C, D)** Cell migration paths of cells in normoxic and hypoxic groups treated with different concentrations of ATRA at 0, 24, 48, and 72 h (The scale in the image is 200 microns); **(E, F, G, H)** Cell apoptosis status of cells in normoxic and hypoxic groups after treatment with different concentrations of ATRA and cultured for 48 h. Quadrant Q1: Necrotic cells (Annexin V-fluorescein isothiocyanate (FITC)–/propidium iodide (PI)+); Q2: Late apoptotic cells (Annexin V-FITC+/PI+); Q3: Viable cells (Annexin V-FITC–/PI–); Q4: Early apoptotic cells (Annexin V-FITC+/PI–). The percentages of cells in Q1, Q2, Q3, and Q4 are shown. The percentage of apoptotic cells was calculated by adding the percentages of cells in Q2 + Q3. Data are presented as mean ± SEM for three independent experiments. **p < 0.01, ***p < 0.001.

The exogenous addition of ATRA showed a dose-dependent regulation of cell proliferation as detected by CCK-8: low concentrations of ATRA (10 nM, 100 nM) promoted cell viability under both normoxic and hypoxic conditions (^***^p < 0.001), while high concentrations of ATRA (1 μM, 10 μM) significantly inhibited cell survival ^(***^p < 0.001; Figures 2A, B). Cell scratch assay further showed that low concentrations of ATRA (10 nM, 100 nM) under normoxic conditions enhanced cell migration ability, while high concentrations of ATRA (1 μM, 10 μM) resulted in diminished migration ability accompanied by cell death (Figure 2C). Cell migration ability was significantly impaired under hypoxic environment, and a large number of cell deaths were observed in the high-concentration ATRA-treated group (Figure 2D). Flow apoptosis analysis showed that 10 nM ATRA decreased the apoptosis rate in normoxic and hypoxic groups, and 10 μM increased the apoptosis rate, the apoptosis rate increased with the increase of ATRA concentration, and this effect was more pronounced under hypoxic conditions. The effects of ATRA on yak subcutaneous adipocytes were concentration- and oxygen-dependent: low concentrations of ATRA exerted positive regulation by promoting survival and migration, while high concentrations of ATRA exacerbated cell damage by inducing apoptosis and inhibiting migration, and the cytotoxicity of high concentrations of ATRA was further amplified by the hypoxic conditions (Figures 2E–H). The cytotoxic effects of high concentrations of ATRA were further amplified under hypoxic conditions. This finding suggests that ATRA may play dual roles in the regulation of adipocyte homeostasis, and its specific molecular mechanism may involve the synergistic effects of oxygen-sensitive signaling pathways and apoptosis-related proteins.

### 3.2 Effects of ATRA on the differentiation ability of yak subcutaneous adipocytes

**Figure 3:**
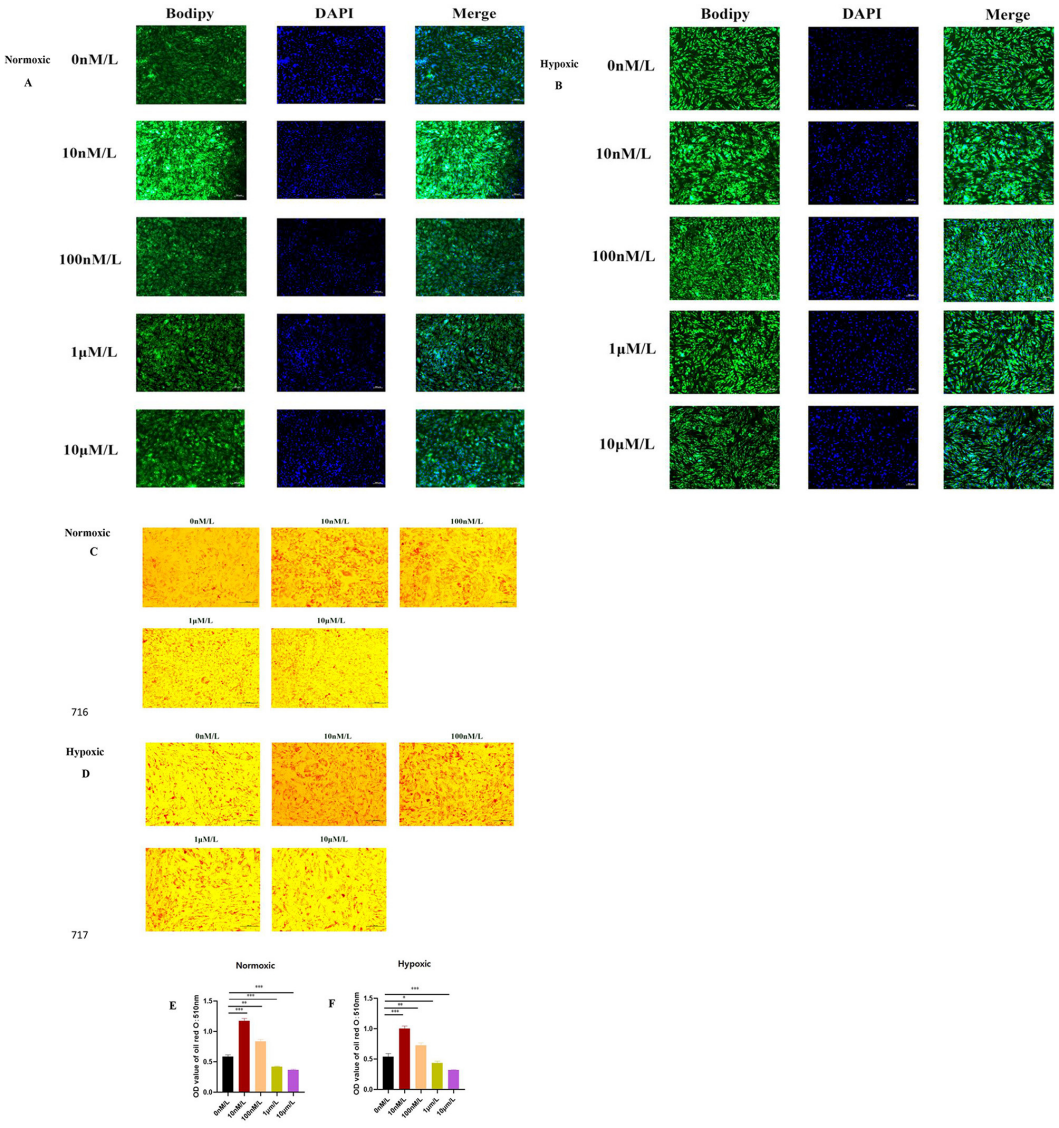
Effects of ATRA on the differentiation ability of yak subcutaneous adipocytes: **(A, B)** Bodipy staining of cells in normoxic and hypoxic groups treated with different concentrations of ATRA and cultured for 48 h; **(C, D)** Oil Red O staining of cells in normoxic and hypoxic groups treated with different concentrations of ATRA and cultured for 48 h, showing lipid droplet deposition; **(E, F)** Quantitative results of Oil Red O staining (OD value 510 nm). Data are presented as mean ± SEM for three independent experiments. Significance levels were defined as *p < 0.05, **p < 0.01, ***p < 0.001, and ns indicates no significant difference.

BODIPY results showed that low concentrations of ATRA (10 nM, 100 nM) partially restored lipid droplet accumulation in the normoxic and hypoxic groups, while high concentrations of ATRA (1 μM, 10 μM) further inhibited lipid droplet formation (Figures 3A, B). The oil red O results (Figures 3C, D) further showed that lipid droplet deposition was more pronounced at 10 and 100 nM ATRA concentrations under both normoxic and hypoxic conditions, and the sensitivity to ATRA was higher in the hypoxic group as revealed by quantitative analysis (Figures 3E, F). The above results suggested that ATRA had a dual effect on the regulation of lipid metabolism in yak subcutaneous adipocytes: low concentration of ATRA maintained the differentiation function of yak subcutaneous adipocytes by promoting the generation of lipid droplets, whereas high concentration of ATRA exacerbated metabolic disorders by inhibiting the deposition of lipids and the inhibitory effect of high concentration of ATRA was significantly enhanced by the hypoxic environment. Combined with the preliminary cell viability and apoptosis data, it was hypothesized that ATRA may affect adipocyte homeostasis under oxygen-dependent conditions by regulating lipid metabolism-related pathways in synergy with apoptosis signaling. In addition, the metabolic inhibitory effect of high concentration of ATRA under hypoxic conditions may be associated with mitochondrial dysfunction or increased endoplasmic reticulum stress, and the specific mechanism needs to be further verified by transcriptomic or proteomic analysis.

### 3.3 Expression of ATRA-related genes

**Figure 4:**
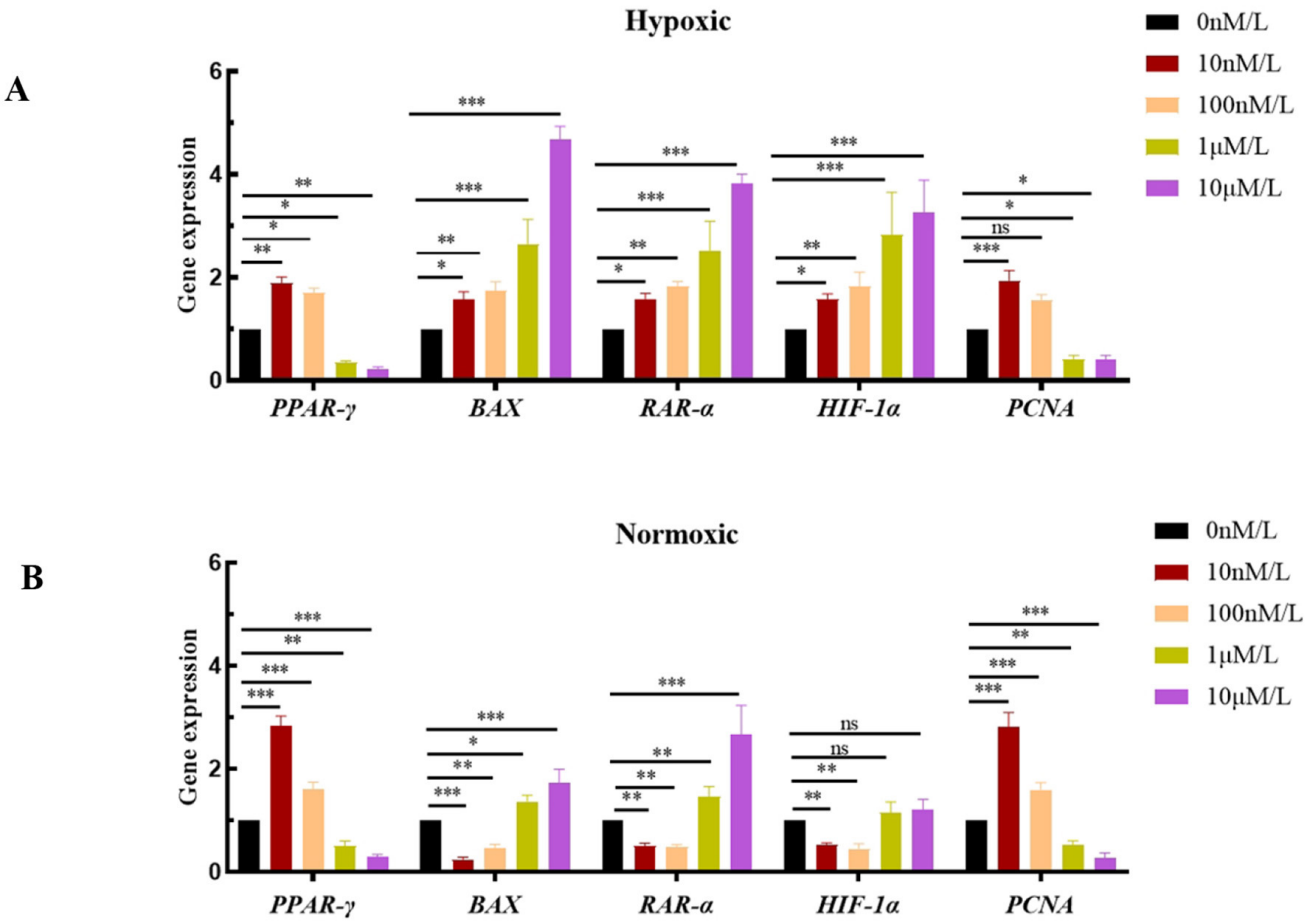
NA expression levels of ATRA-related genes: **(A, B)** mRNA expression levels of different ATRA-related genes in normoxic and hypoxic group cells treated with different concentrations of ATRA and cultured for 48 h. Data are presented as mean ± SEM for three independent experiments. Significance levels were defined as *p < 0.05, **p < 0.01, ***p < 0.001, and ns indicates no significant difference.

In this study, *PCNA, PPAR-*γ, *RAR-*α, *HIF-1*α, and *BAX* were chosen as key target genes due to their core regulatory roles in the ATRA signaling pathway and hypoxic adaptation. qPCR analysis revealed that *HIF-1*α was stably expressed under hypoxic conditions, with its expression level increasing as the ATRA concentration rose (Figure 4A). Meanwhile, low concentrations of ATRA (10 nM, 100 nM) significantly upregulated the mRNA expressions of *PCNA* and *PPAR-*γ in both normoxic and hypoxic groups (Figures 4A, B), promoting the transcriptional activity of genes related to cell proliferation and lipid metabolism. High concentrations of ATRA (1 μM, 10 μM) significantly induced the expression of *RAR-*α and the pro-apoptotic gene *BAX* (Figures 4A, B), and the expression levels of these two genes in the hypoxic group increased significantly with the elevation of ATRA concentration. The aforementioned changes in gene expression indicate that ATRA is involved in the metabolic reprogramming and apoptosis regulation of adipocytes in a hypoxic environment by regulating the *HIF-1*α, *PCNA, PPAR-*γ, and *RAR-*α, *BAX* pathways.

### 3.4 Expression of ATRA-related proteins

**Figure 5:**
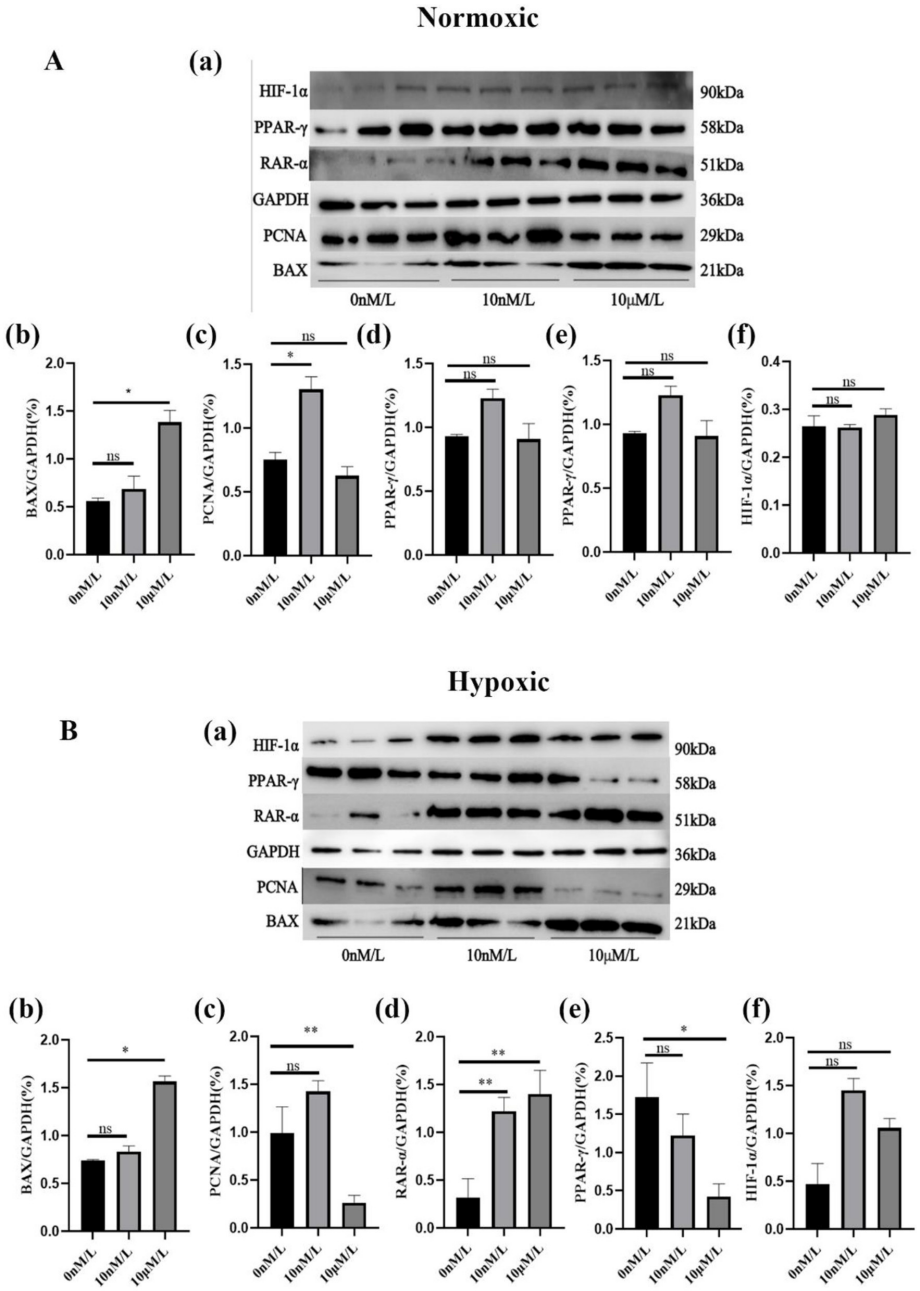
Protein expression levels of ATRA-related proteins: **(A, B)** Expression levels of different ATRA-related proteins in normoxic and hypoxic group cells treated with different concentrations of ATRA and cultured for 48 h. Significance levels were defined as *p < 0.05, **p < 0.01, and ns indicates no significant difference.

When doing Western blot we chose 0 nM, 10 nM, and 10 μM ATRA treated cells to extract proteins for the experiment. the Western blot results showed that the expression of HIF-1α was relatively low under normoxic conditions, and began to be expressed under the lowest oxygen conditions, and there was no too significant difference between the expression at low and high concentrations, which may be due to the threshold effect of the stability of the HIF-1α protein to the change of oxygen concentration. When the oxygen concentration was reduced to a specific threshold, proline hydroxylase activity was significantly inhibited, resulting in the inability of HIF-1α to be degraded via the ubiquitin-proteasome pathway, at which time its protein accumulation had reached a plateau even if the oxygen concentration was further reduced; under hypoxic conditions, a low concentration of ATRA increased the expression of PCNA and PPAR-γ, and a low concentration of ATRA inhibited the expression of PCNA and PPAR-γ expression; regardless of normoxic or hypoxic conditions, high concentration of ATRA could increase the expression of RAR-α and BAX (Figures 5A, B). The above results further verified that ATRA was involved in the metabolic reprogramming and apoptosis regulation of adipocytes under hypoxic environment by regulating HIF-1α, PCNA, PPAR-γ, and RAR-α, BAX pathways.

## 4 Discussion

The unique physiological structure of the yak and its molecular metabolic mechanisms form a highly synergistic low-temperature adaptation strategy. As a typical alpine species, the yak lacks a functional sweat gland system (13), a feature that distinguishes it from lower altitude bovids. Although the absence of sweat glands limits the ability to regulate body temperature through evaporative cooling, at extreme low temperatures between −30 and 40 °C, this “cooling defect” becomes an evolutionary advantage-reducing heat loss through evaporation of water from the surface of the body and forcing yaks to rely on other efficient insulation mechanisms. Anatomical studies have shown that yaks have a skin thickness of 4–6 mm and a SF layer of 10–15 cm, which together with the thick outer layer of hair (20–30 cm) form a three-stage “air-hair-fat” thermal barrier. The thermal resistance of this structure is 3–5 times higher than that of ordinary ruminants, and the core body temperature fluctuation can be controlled within ±1 °C, which greatly reduces the basal metabolic energy consumption in cold environment. Notably, the function of the fat layer is not limited to passive thermal insulation. Uncoupling protein 1 (UCP1) in subcutaneous adipose tissue can achieve non-shivering thermogenesis by mediating proton leakage from the inner mitochondrial membrane and converting the chemical energy generated by oxidative phosphorylation directly into thermal energy (14). This mode of active thermogenesis is functionally complementary to the absence of sweat glands: when the external temperature plummets, adipocytes adapt to the energy constraints of the low-oxygen environment of the plateau by β-oxidatively breaking down the stored triglycerides and utilizing UCP1 to release heat in a directed manner, rather than relying on muscle tremor (which requires large amounts of oxygen consumption) to maintain body temperature. At the molecular metabolic level, the demand for energy conservation driven by sweat gland deficiency may have accelerated the reprogramming of lipid metabolic pathways in yaks.

ATRA, as a lipophilic signaling molecule, plays a multidimensional regulatory role in the proliferation, differentiation, and apoptosis of preadipocytes (15). These physiological processes in animal adipocytes are fundamentally regulated by intracellular signaling networks, intercellular communication, and microenvironmental factors, among which oxygen concentration serves as one of the key environmental variables that can affect the metabolic homeostasis of adipocytes by modulating the ATRA signaling pathway (16).

Safonova et al. (17) first demonstrated ATRA's capacity to promote preadipocyte proliferation and differentiation. Subsequent studies by Lü (18) and Chen et al. (19) revealed that low concentrations of ATRA enhance proliferation in porcine and rat preadipocytes under normoxia (21% O_2_), while higher concentrations suppress proliferation. Based on these studies, we investigated the effects of different concentrations of ATRA on yak subcutaneous adipocytes under normoxic (21% O_2_) and hypoxic (1% O_2_) conditions. Experimental results showed that under hypoxic conditions, the survival rate and migration rate of yak subcutaneous adipocytes were lower than those under normoxic conditions. This adaptive decline may be closely related to the functional activation of HIF-1α, which upregulates the activity of key glycolytic enzymes while suppressing mitochondrial oxidative phosphorylation pathways, forcing cells into a hypoxia-dependent energy metabolism mode (20). Meanwhile, in our study, low concentrations of ATRA (10–100 nM) could partially restore cell viability and mobility, inhibit cell apoptosis, and promote lipid droplet deposition; however, such restoration was slightly less pronounced under hypoxic conditions compared to that under normoxic conditions. The underlying mechanism involves the activation of the PCNA/PPAR-γ signaling axis. Therefore, we analyzed the mRNA and protein expression levels of genes including *PCNA, PPAR-*γ, *RAR-*α, *BAX*, and *HIF-1*α using qPCR and western blot techniques. Among them, *PCNA* plays a crucial role in cell cycle regulation, is closely associated with the proliferation and differentiation of adipocytes, and participates in the regulation of cell growth and metabolic balance (21). *PPAR-*γ is a core regulatory factor for lipid synthesis and storage (22, 23). Under both normoxic and hypoxic conditions, the expression levels of *PCNA* and *PPAR-*γ decreased with the increase of ATRA concentration (0 nM−10 μM), and their overall expression levels under hypoxic conditions were lower than those under normoxic conditions. Western blot results further confirmed that the protein expression levels of *PCNA* and *PPAR-*γ in the hypoxic group were lower than those in the normoxic group, and were positively correlated with the degree of lipid droplet deposition. *RAR-*α is an important subtype of retinoic acid receptors (RARs) in the Nuclear Receptor Superfamily (24). Under both hypoxic and normoxic conditions, low concentrations of ATRA could reduce its expression level, while high concentrations of ATRA could increase its expression level, showing a similar trend. This further indicates that as a key nuclear receptor for ATRA, the bidirectional changes in *RAR-*α expression with ATRA concentration may be the molecular basis for ATRA to achieve dose-dependent regulation. When low concentrations of ATRA were added, the decreased expression of *RAR-*α might reduce its direct regulation of the PCNA/PPAR-γ signaling axis, thereby facilitating *PCNA* and *PPAR-*γ to exert their roles in promoting cell proliferation and lipid droplet deposition, so as to meet the energy storage needs under hypoxic environments. In contrast, high concentrations of ATRA induced the upregulation of *RAR-*α expression, which might enhance its interaction with other signaling molecules. On one hand, it might inhibit adipocyte proliferation and lipid droplet deposition by antagonizing the PCNA/PPAR-γ signaling axis; on the other hand, it might be associated with the upregulation of the pro-apoptotic gene *BAX*, collectively mediating the tendency of cell apoptosis under high concentrations of ATRA. High concentrations of ATRA (1–10 μM) significantly induced BAX-dependent apoptosis and inhibited lipid droplet accumulation, with these effects being more pronounced under hypoxic conditions. This likely reflects an evolutionarily conserved “metabolic checkpoint” mechanism (25), where the pro-apoptotic protein BAX triggers caspase cascade reactions via mitochondrial membrane potential collapse (26), thereby eliminating functionally impaired cells to maintain tissue homeostasis.

Under hypoxic conditions, HIF-1α activates PDK1 to inhibit pyruvate entry into the tricarboxylic acid cycle, thereby enhancing glycolytic energy production (27). In this study, under normoxic conditions, HIF-1α expression was extremely low at both gene and protein levels. In contrast, HIF1-α was stably expressed under hypoxic conditions, and low concentrations of ATRA (10–100 nM) significantly suppressed hypoxia-induced *HIF-1*α mRNA expression and protein accumulation compared to high-concentration ATRA (1 μM−10 μM) treatment groups. This bidirectional regulatory phenomenon suggests that ATRA modulates HIF-1α in a concentration-dependent manner, with potential mechanisms involving interactions between multiple signaling pathways. Low concentrations of ATRA may inhibit HIF-1α transcriptional activity by competitively binding to its coactivators or promoting HIF-1α degradation via the RAR-α-dependent ubiquitin-proteasome pathway (28, 29). However, the promotional effect of high-concentration ATRA on HIF-1α may be related to compensatory feedback mechanisms. When ATRA concentrations exceed metabolic thresholds, mitochondrial reactive oxygen species (ROS) bursts activate the NF-κB pathway or enhance HIF-1α promoter activity through epigenetic remodeling, thereby reversing its inhibitory effect (30).

From an evolutionary physiology perspective, adipocyte responses to ATRA may also exhibit species specificity. Xu et al. (31) demonstrated that ATRA concentrations of 0.2–20 nM inhibit the proliferation of subcutaneous preadipocytes in Holstein cows. Kim et al. (32) investigated the effects of ATRA on *in vitro* adipogenesis using chicken embryonic day 14 (E14) leg-derived preadipocytes and embryonic day 5 (E5) fibroblasts (CEFs). After 48 h of treatment with 0, 100, or 150 μmol/L ATRA, they observed dose-dependent increases in lipid droplet accumulation and significant upregulation of adipogenic marker genes, fatty acid transporters (CD36), and triacylglycerol synthases (DGAT1). In contrast, our study found that 10–100 nM ATRA promoted yak adipocyte proliferation, differentiation, and lipid droplet deposition, while 1–10 μM ATRA suppressed these processes. This discrepancy may arise from plateau-specific natural selection-driven remodeling of metabolic pathways. The yak PPAR-γ promoter region may contain unique hypoxia-responsive elements (HREs), rendering it more susceptible to activation by the ATRA/RAR-α complex under hypoxia. Concurrently, elevated expression of retinoic acid-binding protein CRABP2 enhances ATRA transport efficiency to nuclear receptors (33), while high expression of UCP1 in subcutaneous adipose tissue (34) reduces oxidative phosphorylation-derived ROS accumulation via uncoupling thermogenesis. These adaptive traits collectively constitute yak strategies for balancing energy supply and oxygen utilization efficiency—prioritizing SF storage and insulation while avoiding excessive energy consumption.

Although this study preliminarily elucidates ATRA's regulatory network in hypoxic adaptation, several limitations remain. First, existing *in vitro* models can simulate basic hypoxic microenvironments but fail to fully replicate complex *in vivo* neuroendocrine regulatory processes. For example, the spatiotemporal specificity of paracrine signaling by adipokines such as leptin and adiponectin across tissues has not been integrated into the current experimental system (35). Second, while the study focuses on the overall impact of ATRA on metabolic reprogramming, it lacks in-depth analysis of mitochondrial dynamics and key regulatory nodes in autophagy pathways. Additionally, the exclusive use of male yak samples neglects potential influences of sex differences and seasonal variations on fat metabolism, potentially limiting the ecological applicability of conclusions. To address these limitations, future studies should combine single-cell transcriptomics to systematically map heterogeneous responses of adipocytes to ATRA treatment and employ CRISPR/Cas9 gene editing to functionally validate core molecules in the PCNA/PPAR-γ/RAR-α/BAX/HIF-1α pathway. *In vivo* ATRA intervention experiments in yaks should comprehensively evaluate effects on SF deposition and cold stress tolerance while exploring synergistic interactions between ATRA and other hypoxia adaptation factors such as erythropoietin and vascular endothelial growth factor (VEGF). Establishing cross-sex and cross-season dynamic research models will help elucidate metabolic regulation mechanisms under hormone cycle-environmental factor interactions. Through multi-scale integrative analysis, the theoretical framework for plateau animal hypoxic adaptation can be further refined.

## 5 Conclusion

This study confirms that ATRA profoundly influences the hypoxic adaptability of yak subcutaneous adipocytes through dose-dependent regulation of the PCNA/PPAR-γ/RAR-α/BAX and HIF-1α signaling networks. This process not only involves the dynamic balance of metabolic reprogramming and cell fate but also reflects the unique survival strategies formed by plateau species during long-term natural selection. Future research needs to integrate multi-omics and *in vivo* models to further reveal the global characteristics of the ATRA regulatory network, providing theoretical support for plateau biology and innovations in animal husbandry.

## Data Availability

The datasets presented in this study can be found in online repositories. The names of the repository/repositories and accession number(s) can be found in the article/supplementary material.
